# Two new species of *Chaco* Tullgren from the Atlantic coast of Uruguay (Araneae, Mygalomorphae, Nemesiidae)

**DOI:** 10.3897/zookeys.337.5779

**Published:** 2013-10-01

**Authors:** Laura Montes de Oca, Fernando Pérez-Miles

**Affiliations:** 1 Sección Entomología, Facultad de Ciencias, Universidad de la República. Iguá 4225. CP 11400. Montevideo, Uruguay; 2 Laboratorio de Etología, Ecología y Evolución, Instituto de Investigaciones Biológicas Clemente Estable. Av. Italia 3318. CP 11600. Montevideo, Uruguay

**Keywords:** Spiders, Taxonomy, Cladistics, *Chaco*, Nemesiidae, Natural history

## Abstract

We describe two new species of the nemesiid spider genus *Chaco* from Rocha Province, Uruguay. These new species are diagnosed based on genital morphology, male tibial apophysis spination, and burrow entrance. We test cospecificity of one species, *Chaco costai*,via laboratory mating experiments. The new species are diagnosed and illustrated and habitat characteristics, and capture behavior are described. We conduct a cladistic analysis based on a previously published morphological character matrix that now includes the newly described species.

## Abstract (es)

En este trabajo se describen dos nuevas especies de arañas, nemésidas del género *Chaco* recolectadas en el departamento de Rocha, Uruguay. Las nuevas especies se distinguen de las conocidas por caracteres tomados de la morfología de la genitalia, número de espinas en la apófisis tibial de los machos y la tapa-cortina en la cueva. Se realizó un análisis filogenético de las especies del género *Chaco* incluyendo las nuevas especies en una matriz previa.

## Introduction

The family Nemesiidae Simon, 1889 comprises 43 genera (Platnick 2010) of medium sized spiders that have a transverse fovea, eyes grouped on a tubercle, 2-4 short spinnerets, anterior tarsi without spines, tarsi III and IV with light or absent scopula, without claw tufts and superior tarsal claws bipectinate with numerous teeth. Several authors have questioned nemesiid monophyly (Raven 1985, Goloboff 1993, 1995, Hedin and Bond 2006). Although morphological characters are somewhat equivocal, molecular data seem to support the group (Bond and Hedin 2006, Hedin and Bond 2006, Bond et al. 2012). Goloboff (1995) conducted the last major revision of the South American species of this family; since, only a few contributions to the systematics of the Nemesiidae family have been made (Indicatti and Lucas 2005; Indicatti et al. 2008; Lucas et al. 2008; Lucas and Indicatti 2010). Nemesiids live in silk tubes under stones or logs, or in burrows that are covered by a trapdoor (Goloboff 1995). In Uruguay the family is represented by four genera: *Acanthogonatus* Karsch, 1880; *Pycnothele* Chamberlin, 1917; *Stenoterommata* Homberg, 1881 and *Chaco* Tullgren, 1905. Due to a number of life history characteristics, they are difficult to collect and consequently little is known about their biology. Montes de Oca and Pérez-Miles (2003) reported the presence of *Chaco* Tullgren, 1905 in Uruguay but did not identify the species.

The genus *Chaco* was originally described on the basis of the type species *Chaco obscura* Tullgren, 1905 known from a female specimen. The species was characterized as having posterior lateral spinnerets with two apical segments of equal length and the absence of labial cuspules (Tullgren 1905). Goloboff (1987, 1995) diagnosed *Chaco* using the following character combination: four short spinnerets, eight eyes grouped on a tubercle, anterior legs with few spines, anterior tarsi scopulate, without spines and no claw tufts; tarsal claws with numerous teeth in two rows. Males of the genus can be distinguished by having a distal prolateral spur on tibia I comprising three or more spines, absence of inferior claw on all tarsi, patella III with 1-1-1 spines and anterior tibia without scopula. Females of *Chaco* are distinguished from other nemesiid genera by the absence of the following features: ITC from all legs; spigots with pumpkin-like socket; scopula on the anterior tibia (present in *Prorachias* and *Pselligmus*) and patella IV spination. Additionally *Chaco* females lack the autapomorphies indicated for the Diplothelopsini: anterior median eyes much larger than the minute posterior median eyes, posterior eye row slightly recurved, and short, wide caput (Goloboff 1995).

The characteristics of some individual specimens discovered along the coast of Uruguay have the diagnostic characteristics described for *Chaco* but differ from all the known species. In this article we describe, diagnose, and illustrate two new species: *Chaco castanea* sp. n. and *Chaco costai* sp. n. We present a cladistic reanalysis of the genus with newly described species and present some natural history data for the new taxa.

## Material and methods

Specimens were examined using an Olympus SZH stereomicroscope. The description of color was based on live organisms when possible. Abbreviations: AME anterior median eyes, ALE anterior lateral eyes, PME posterior median eyes, PLE posterior lateral eyes, OQ ocular quadrangle, P prolateral, R retrolateral, D distal, STC superior tarsal claw, ITC inferior tarsal claw, FCE-MY Collection of Facultad de Ciencias, Entomología – Mygalomorphae. All measurements are in mm and were taken with an ocular micrometer. Total body length excludes chelicerae and spinnerets. Lengths were measured along a dorsal longitudinal line and widths were taken at maximum values. The OQ length was measured from the anterior edge of ALE to the posterior edge of PLE; the sternum length from the posterior angle to the labium edge (Goloboff 1995). Terminology for spination is modified from Pérez-Miles and Weinmann 2010. The formula gives the number of spines in the following order: dorsal–prolateral–retrolateral–ventral (p indicates a pair of spines that occur at this position). Spermathecae were cleared with clove oil and illustrated in dorsal view. Left male palpal bulb was removed from the cymbium and illustrated in prolateral and retrolateral views. Specimens were photographed using a Lumenera INFINITY 3 camera.

**Cladistic analysis.** We scored the newly described species (*Chaco castanea* and *Chaco costai*) for 32 characters (Table 1) from Goloboff (1995), removing invariant characters and adding five new ones (characters 27–31). Maxillary cuspules were codified separately for males and females (characters 9 and 10) to account for sexual dimorphism. The ingroup comprised 9 taxa: *Chaco castanea* sp. n.; *Chaco costai* sp. n.; *Chaco obscura* Tullgren, 1905; *Chaco patagonica* Goloboff, 1995; *Chaco sanjuanina* Goloboff, 1995; *Chaco socos* Goloboff, 1995; *Chaco tecka* Goloboff, 1995; *Chaco tigre* Goloboff, 1995 and *Chaco tucumana* Goloboff, 1995. The outgroup included: *Chilelopsis calderoni* Goloboff, 1995; *Diplothelopsis bonariensis* Mello-Leitão, 1938 and *Lycinus longipes* Thorell, 1894. The outgroup taxa are considered close relatives of *Chaco* (Goloboff, 1995). The tree was rooted using *Lycinus longipes* Thorell, 1894. *Chaco melloleitaoi* (Bücherl, Timotheo & Lucas, 1971) is not included in the analysis because it in fact belongs to a different genus (Indicatti et al. 2011). It differs from *Chaco* by the presence of two prolateral megaspines on male first tibia and in the palpal organ morphology.

**Table 1.** Data matrix for the genus *Chaco* and the outgroup.

											**1**										**2**										**3**	
	**0**	**1**	**2**	**3**	**4**	**5**	**6**	**7**	**8**	**9**	**0**	**1**	**2**	**3**	**4**	**5**	**6**	**7**	**8**	**9**	**0**	**1**	**2**	**3**	**4**	**5**	**6**	**7**	**8**	**9**	**0**	**1**
*Lycinus longipes*	0	1	1	2	0	0	0	0	0	0	0	0	2	1	0	1	1	1	1	0	0	0	1	1	0	0	0	0	0	1	0	?
*Diplothelopsis bonariensis*	0	1	1	2	0	0	0	0	0	0	0	1	2	1	?	1	0	1	1	0	0	0	1	0	0	0	0	?	0	0	0	?
*Chilelopsis calderoni*	0	1	0	1	1	0	0	1	2	2	0	0	1	0	0	1	0	1	0	0	0	0	0	2	1	0	0	1	0	0	0	?
*Chaco obscura*	0	0	0	1	1	0	1	0	1	1	1	1	1	0	0	1	0	0	0	0	0	1	1	1	1	0	1	0	0	0	0	0
*Chaco tucumana*	0	0	0	1	1	0	1	0	2	1	1	1	1	0	0	1	0	0	0	0	0	1	1	1	1	0	1	0	0	0	0	0
*Chaco socos*	1	0	0	1	1	0	1	0	2	2	1	0	1	0	1	1	1	0	0	0	1	1	1	1	1	1	1	1	0	0	0	0
*Chaco tigre*	1	0	0	1	1	0	1	0	1	1	1	0	1	0	1	1	0	0	0	0	1	1	1	1	1	1	1	0	0	0	0	1
*Chaco patagonica*	0	0	0	0	1	1	0	1	0	?	1	0	0	?	1	1	?	?	0	1	1	1	?	?	?	1	0	0	0	?	?	?
*Chaco tecka*	0	0	1	1	1	1	1	1	1	?	1	0	0	?	1	1	?	?	0	0	1	1	?	?	?	1	0	1	0	?	?	?
*Chaco sanjuanina*	0	0	0	0	1	1	0	1	1	1	1	0	0	0	1	1	0	0	0	1	1	1	0	1	0	1	?	0	0	0	0	1
*Chaco castanea*	0	0	0	1	1	1	0	0	2	1	1	1	1	0	1	0	0	0	0	0	0	1	1	1	1	0	?	2	0	0	0	1
*Chaco costai*	0	0	0	1	1	0	0	0	2	2	1	0	1	0	0	0	0	0	0	0	1	1	1	1	1	0	0	0	1	1	1	0

The data matrix (Table 1) was constructed using Nexus Data Editor ver 0.5.0 software (Page 2001). The cladistic analysis was carried out with the program TNT version 1.1 (Goloboff et al. 2003), using maximum parsimony as the optimality criterion. Tree searches were conducted using implicit enumeration and implied weighting (Goloboff 1993) with concavity indices (k) ranging from 1 to 6.

**Characters scored**. (0) Clypeus: 0, wide; 1, narrow (1) PE row: 0, recurved; 1 procurved (2) Eyes: 0, AME and PME subequal size; 1, AME much larger than PME (3) Pubescence: 0, absent; 1, light; 2, dense (4) Sternum: 0, wide; 1, normal; 2, narrow (5) Sternal sigilla: 0, conspicuous; 1, inconspicuous (6) Leg color: 0, uniform; 1, patterned (7) Setae on female posterior legs: 0, normal; 1, dense (8) Maxillary cuspules in females: 0, few (0-10); 1, medium (11-30); 2, many (over 30) (9) Maxillary cuspules in males: 0, few (0-10); 1, medium (11-30); 2, many (more than 30) (10) Rastellum: 0, weak; 1, strong (11) Female tarsi: 0, rigid; 1, flexible (12) Scopula IV: 0, absent/ very light; 1, light; 2, dense (13) Trichobothria on male cymbium: 0, medial third; 1, basal half (14) PMS spigot number: 0, many; 1, few (15) Male metatarsus IV: 0, 1-1-1P SUP; 1, 0-0-1P SUP (16) Dorsal spines in male palpal tibia: 0, absent; 1, present (17) Spines on male patella I-II: 0, 0/1P; 1, 1-1-1P (18) Female patella IV: 0, 0/1P; 1, 1-1-1P (19) Spines on female tarsi IV: 0, absent; 1, present (20) Spines on female tibia/metatarsus I: 0, short; 1, long (21) Male tibial spur: 0, absent; 1, present (22) Male palpal tibia: 0, short; 1, long (23) Male bulb keels: 0, absent; 1, parallel keels or ridges along embolous base; 2, lateral keels or flanges (24) Male bulb duct: 0, basal portion evenly curved; 1, basal portion strongly sinuous (25) Female spermathecae: 0, no basal sphere; 1, with basal sphere (26) Habits: 0, flap door; 1, trap door. (27) Spermathecae fundus 0, subspherical; 1, reniphorm (28) female tibiae 0, normal; 1, short (29) Setae on male cymbium 0, thin hair like setae; 1, thickened setae (30) Two long dorsal setae on palpal tibiae setae 0, absent; 1, present (31) Spines on male tibial apophysis 0, five or less; 1, more than 5.

## Results and discussion

### Cladistic results

Analysis of the morphological data using implied weighting and implicit enumeration resulted in 2 most parsimonious trees. Topologies were stable across K values 1-6 (62 steps, CI = 0.61, RI = 0.65, K = 1, fit = 8.75, K = 6, fit = 2.79; Fig. 5A-B). The genus *Chaco* was recovered as monophyletic including the two new species, supported by 4 characters: PE row procurved (1), strong rastellum (10), spination of male patellae I-II (17) and the presence of male palpal tibial spur (21). The main difference between the topology of the two recovered trees was: *Chaco socos* + *Chaco tigre* is the sister group of *Chaco obscura* + *Chaco tucumana* in one tree, while in the other *Chaco socos* + *Chaco tigre* is the sister group of (*Chaco teka* (*Chaco patagonica*, *Chaco sanjuanina*)). The consensus tree (Fig. 5C) recovers a polytomy for *Chaco castanea*, *Chaco obscura*, *Chaco tucumana*, and the clades (*Chaco obscura*, *Chaco tucumana*) and (*Chaco teka* (*Chaco patagonica*, *Chaco sanjuanina*)). Based on these data the monophyly of the genus *Chaco* appears to be well supported with the inclusion of the new species; the addition of new characters in the future will be necessary to improve the resolution of relationships among several species. Regarding the biology of *Chaco costai*, the flap-like door of the burrow may be explained as an adaptation to sandy soil habitat.

### Natural History

*Chaco costai* specimens are typically found in sandy soils of oceanic and river coastal areas associated with psammophyte vegetation (Fig. 2F). Individuals were collected from tubular vertical burrows of about 100mm length; the entrance diameter is about 10mm. The spider closes the burrow entrance with sand and silk when disturbed. *Chaco patagonica*, *Chaco costai* make a burrow that is covered with a thin, flaplike door (Fig. 2E). The door actually consists of a prolongation of the silk layer lining the interior of the burrow, covered by grains of sand; it is flexible and loosely articulated. According to field and laboratory observations, the spider begins foraging at night by standing at the top of the burrow with legs I-III extended lying in the substrate (Fig. 2E); similar to that reported by Bond and Coyle (1995) for *Ummidia*. After a prey item is captured, the spider returns to the interior of the burrow to feed; the burrow entrance remains open until later when the spider returns to the entrance to repair and close the flap-like- door.

A copulation event observed in the laboratory occurred over an eight minute time period at 18 °C. The male appeared to initiate courtship with body vibrations and pulling silk threads with his chelicerae. Body vibrations were caused by spasmodic contraction of legs I and II. The male approached the female burrow entrance and opened it with his chelicerae; the female then emerged from the burrow. Copulation took place at the burrow entrance; the male clasped his tibial apophysis with female chelicerae. The male performed 23 palpal insertions, alternating right and left palps. The mean duration of the insertions was 21.09±12.73 seconds. After copula the male retreated with legs I extended and female retreated in the burrow but maintained her first legs out towards the entrance. After 17 minutes the female closed the burrow flap-door.

## Taxonomy

### Family Nemesiidae Simon, 1889
Genus *Chaco* Tullgren, 1905

#### Chaco castanea sp. n.

http://zoobank.org/42AA4036-8DA2-4C0C-8AAB-92B5A15649DC

http://species-id.net/wiki/Chaco_castanea

Figs 1A–D , 3A–E

##### Types.

Male holotype (deposited in FCE-MY 0767) from, Rocha, Perla de Rocha, 34°25.0'S, 53°51.0'W, i.2001, coll. G. Calixto Female paratype (deposited in FCE-MY 0770) from Rocha, Cabo Polonio, 34°24.0'S, 53°47.0'W, 24.i–18.iii.2003, coll. F. Achaval. **Additional material examined.** Male from Rocha, Cabo Polonio, 19.xii.2003–18.iii.2004, coll. F. Achaval. 1m (deposited in FCE-MY 0769), Female from Rocha, Perla de Rocha, 34°25.0'S, 53°51.0'W, i.2001, coll. G. Calixto, 1f (FCE-MY 0766), female from Rocha, Cabo Polonio, 34°24.0'S, 53°47.0'W, 18.i–18.iii.2005, coll. F. Achaval, 1f (deposited in FCE-MY 0797).

##### Etymology.

The specific epithet is a noun taken in apposition (chestnut) and is in reference to the brownish coloration of this species.

**Figure 1.**
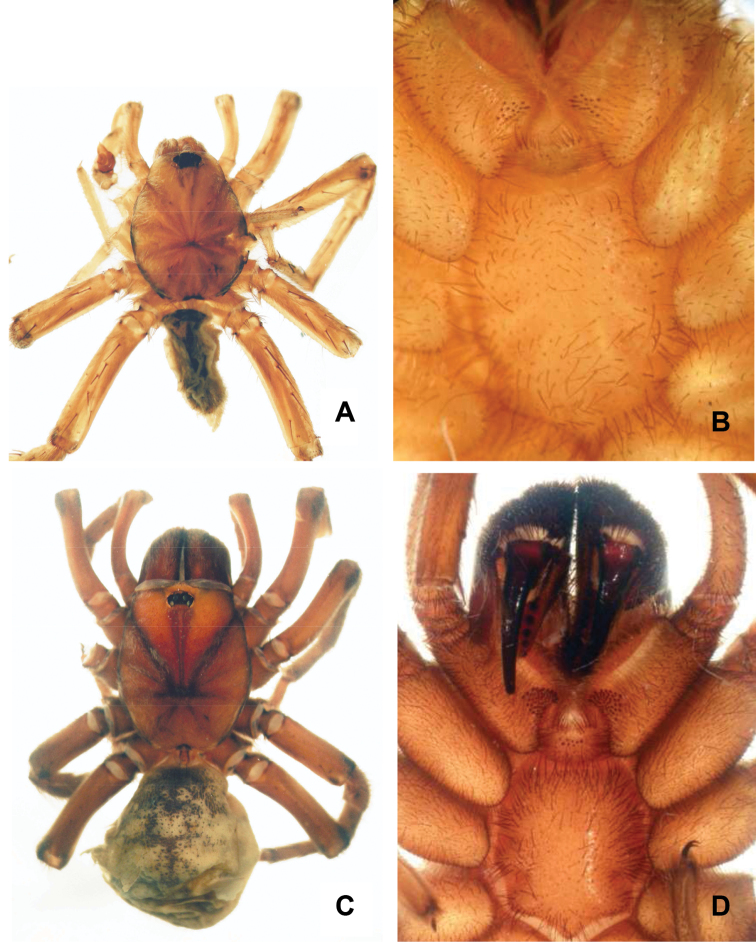
*Chaco castanea*. **A** Male holotype habitus, dorsal view **B** Male holotype maxillae and labium showing cuspules **C** Female paratype habitus, dorsal view **D** Female paratype maxillae and labium showing cuspules.

**Diagnosis.** Males (Fig. 1A) uniquely possess a tibial apophysis with 4 spines (Fig. 3D). *Chaco castanea* males differ from *Chaco tigre* and *Chaco socos* males by having a palpal organ with a sinuous spermophor and parallel longitudinal ridges (Figs 3A–C), and by having a PME and AME that are subequal in diameter. Females of *Chaco castanea* (Fig. 1C) differ from the other known *Chaco* species by the presence of a large reniform spermathecal receptacle in combination with a short sinuous duct (Fig. 3E).

**Figure 2.**
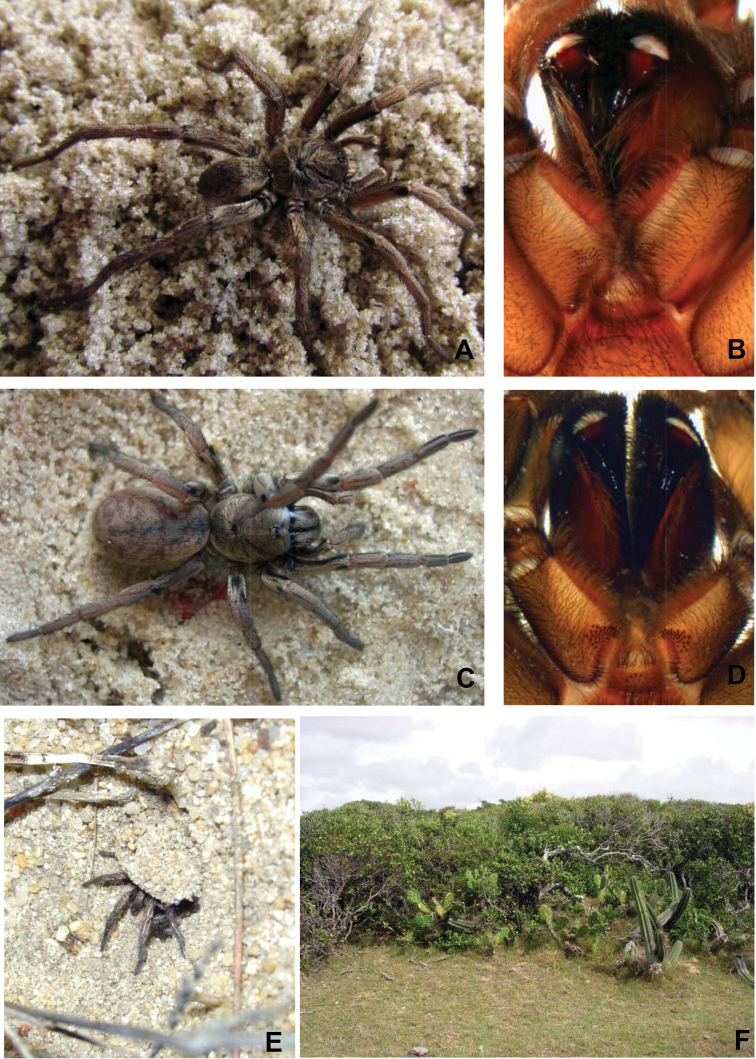
*Chaco costai*. **A** Male habitus, dorsal view **B** Male maxillae and labium showing cuspules **C** Female habitus, dorsal view **D** Female maxillae and labium showing cuspules **E** *Chaco costai* female ambushing in the burrow entrance, see the flap-like door **F** Habitat of *Chaco costai* showing psammophyte vegetation.

**Figure 3.**
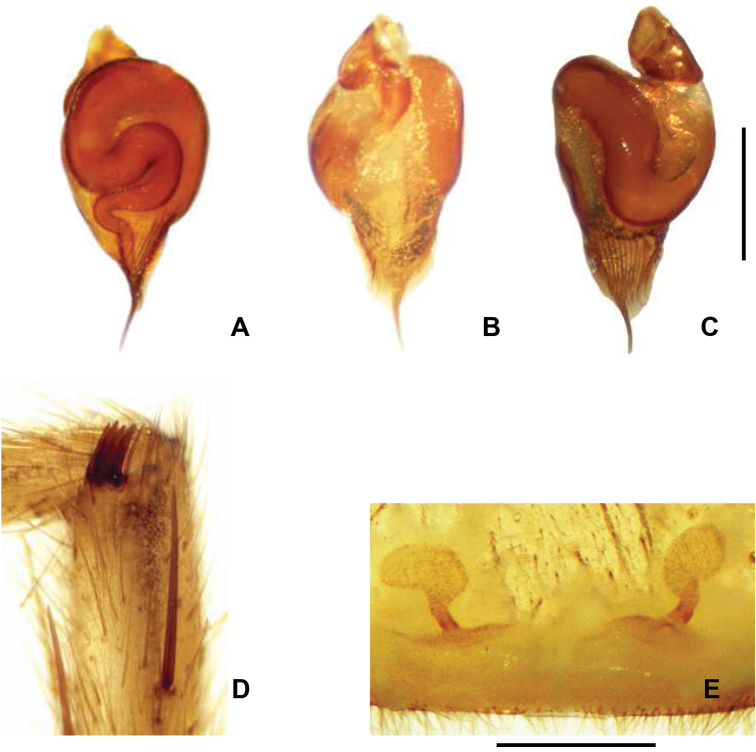
*Chaco castanea*. **A** Palpal organ, dorsal view **B** Palpal organ, retrolateral view **C** Palpal organ prolateral view **D** Male tibial apophysis with 4 spines, prolateral view **E** Spermathecae, dorsal view. Scale = 5mm.

##### Description.

Male (holotype). Total length 7.75; carapace 4.13 long, 3.27 wide; eight eyes grouped on a tubercle, AME 0.17; PME 0.13; ALE 0.16; PLE 0.16; OQ 0.86 long, 0.44 wide; clypeus 0.06; fovea 0.63; sternum oval, 1.93 long, 1.56 wide. Posterior sigillae sub-circular, sub-marginal. Labium sub-rectangular, 0.2 long, 0.56 wide; labial cuspules absent; 18/20 maxillary cuspules (Fig. 1B); chelicerae with 7 promarginal teeth of similar size; rastellum with 26 short, thick conical setae on promargin narrowing through retromargin. Leg, palpal measurements in Table 2; chaetotaxy in Table 3. Tarsus I-IV scopula entire; metatarsi I-II distal third, III-IV absent. Anterior tibiae without scopula. STC with numerous teeth in two lateral rows. ITC I-IV absent. Claw tufts absent. Tibial apophysis with 4 prolateral apical spines (Fig. 3D). Palpal organ spermophor strongly sinuous (Figs 3A–C). Four spinnerets, PMS short monoarticulated, PLS triarticulated apical article short, domed. Spigots without pumpkin-like socket. Body, legs light brown, abdomen with dark brown spots.

Female (paratype). Total length 18.2; carapace 7.2 long, 5.6 wide; caput raised; eight eyes grouped on a tubercle, AME 0.24; PME 0.25; ALE 0.36; PLE 0.31; OQ 0.75 long, 1.6 wide; clypeus 0.14; fovea 0.9; sternum oval, 2.3 long, 2.2 wide. Posterior sigillae sub-circular, sub-marginal. Labium sub-rectangular, 0.7 long, 1.3 wide; 1 labial cuspule; maxillary cuspules 48/62 (Fig. 1D). Chelicerae with 6 promarginal teeth; first tooth smaller than second, decreasing thereafter; 10 retromarginal denticles; rastellum with 45 short, thick conical setae on promargin. Leg, palpal measurements in Table 4; chaetotaxy in Table 5. Tarsus I-IV scopula entire, metatarsus I-II entire, III–IV absent. Anterior tibiae without scopula. STC with numerous teeth in two lateral rows. ITC I-IV absent. Claw tufts absent. Palpal claw with 4 teeth in prolateral median line. Two spermathecal receptacles, single sinuous neck; reniform fundus (Fig. 3E). Four spinnerets, PMS short monoarticulated, PLS triarticulated apical article short, domed. Spigots without pumpkin-like socket. Coloration as in male.

**Table 2.** Length of legs palpal segments of the holotype male of *Chaco castanea*.

	**Fe**	**Pa**	**Ti**	**Mt**	**Ta**	**Total**
Palp	2	0.83	1.71	–	0.83	5.37
I	3.4	2	2.53	2.67	2.07	12.67
II	3.27	1.67	0.87	2.67	2.2	10.68
III	3	1.45	2.1	3.17	2.43	12.15
IV	3.93	1.6	3.73	4.33	2.93	16.52

**Table 3.** Spination of legs and palps of holotype male *Chaco castanea*.

	**Fe**	**Pa**	**Ti**	**Mt**	**Ta**
Palp	2-1-1-0	0	0-3-1-0	–	0
I	4-1-1-0	0	0-2-0-6	0-1-1-3	0
II	5-2-3-0	0-1-0-0	0-2-0-3	0-1-1-3	0
III	0-0-0-6	0-3-1-0	6-2-2-6	3-4-4-6	0
IV	6-6-3-0	0-1-1-0	3-2-3-6	3-3-2-6	0

**Table 4.** Length of legs and palpal segments of the paratype female of *Chaco castanea*.

	**Fe**	**Pa**	**Ti**	**Mt**	**Ta**	**Total**
Palp	3.6	2.1	2.2	–	2.2	10.1
I	4.7	2.8	3.1	2.8	2.0	15.4
II	4.2	2.7	3.0	2.8	1.9	14.6
III	3.6	2.4	2.3	3.5	2.4	14.2
IV	5.0	3.2	4.2	5.1	2.9	20.4

**Table 5.** Spination of legs and palps of paratype female *Chaco castanea*.

	**Fe**	**Pa**	**Ti**	**Mt**	**Ta**
Palp	0-1-0-0	0-1-0-0	0-4-1-6	–	0
I	0-1-0-0	0-0-0-0	0-0-0-1	0-0-0-3	0
II	0	0-2-0-0	0-2-0-2	0-1-0-2	0
III	0-1-0-0	0-3-1-0	1-2-2-2	2-2-3-6	0
IV	1-0-1-0	0-0-0-0	0-2-3-6	0-2-3-8	0

##### Distribution.

Uruguay, Rocha, Perla de Rocha and Cabo Polonio.

#### Chaco costai sp. n.

http://zoobank.org/65418A73-632C-4AEF-8CE9-2740E0F09EC4

http://species-id.net/wiki/Chaco_costai

Figs 2A–F , 4A–E

##### Types.

Male holotype (FCE-MY 1007) female paratype (FCE-MY 1006) and from Rocha, Perla de Rocha, 34°25.63'S, 53°52.27'W, 26-28.xii.2011, coll. A. Laborda, C. Castro, L. Montes de Oca.

##### Etymology.

The specific epithet is a patronym in honor of Fernando G. Costa, a recognized Uruguayan arachnologist who greatly contributed to the knowledge of spiders and has inspired many colleagues and students.

##### Diagnosis.

**Figure 4.**
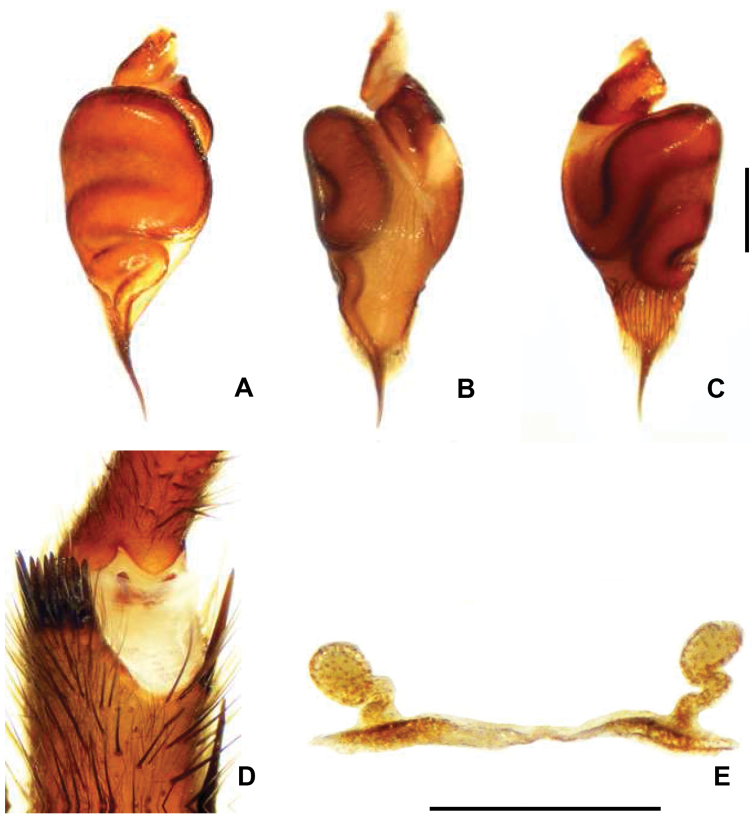
*Chaco costai*. A Palpal organ, dorsal view **B** Palpal organ retrolateral view **C** Palpal organ prolateral view **D** Male apophysis with 10 spines, prolateral view **E** Spermathecae, dorsal view. Scale = 5mm.

Males of *Chaco costai* (Fig. 2A) differ from the other species of the genus, except *Chaco obscura*, by the presence of numerous spines (7–10) on tibial apophysis (Fig. 4D); they can be distinguished from *Chaco obscura* by having a shorter embolous (Figs 4A–C). Female *Chaco costai* specimens (Fig. 2C) differ from most species of *Chaco* by having spermathecae with a sinuous neck (Fig. 4E). The species is distinguished from the geographically proximate species *Chaco castanea* by having a longer spermathecal neck and from *Chaco obscura* by the sinuous neck. *Chaco costai* differ from all other species of the genus (except *Chaco patagónica* and *Chaco tecka*) by having a flap door that covers the burrow (Fig. 2E).

**Figure 5.**
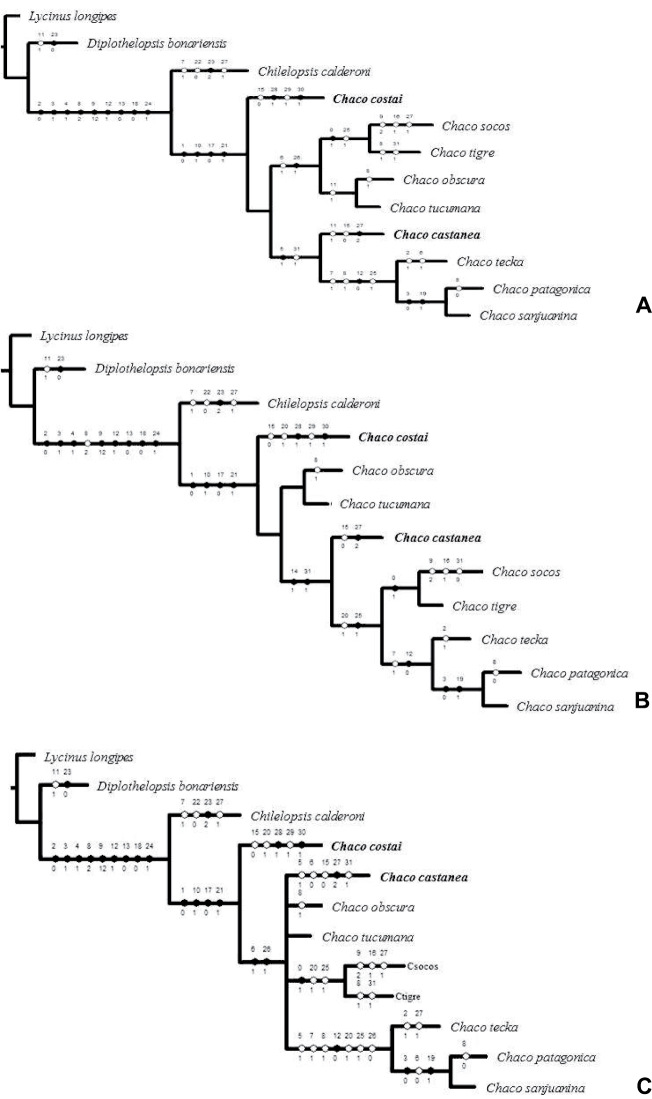
Results from cladistics analyses. **A–B** Most parsimonious trees obtained by TNT (implied weighting). Length = 62, CI = 0.61, RI = 0.65, K =1 Fit = 10.33, K = 6 Fit = 3.48 **C** Strict consensus of cladograms **A** and **B**.

##### Description.

Male (holotype). Total length 14.9; carapace 7.7 long, 6.2 wide; eight eyes grouped on a tubercle, AME 0.28; PME 0.23; ALE 0.38; PLE 0.36; OQ 0.79 long, 1.04 wide; clypeus 0.22; fovea transverse, slightly procurve 1.5; sternum oval, 3.1 long, 2.8 wide. Posterior sigillae sub-circular, sub-marginal. Labium sub-rectangular, 1.2 long, 3.5 wide; 4 labial cuspules; 42/38 maxillary cuspules (Fig. 2B); chelicerae with 2 row of teeth, 6 promarginal, 5 retromarginal denticles; rastellum with 16 short, thick conical setae on promargin. ITC absent. Claw tufts absent. Leg, palpal measurements in Table 6; chaetotaxy in Table 7. Tarsus I-III scopula entire, IV divided; metatarsi I 4:5D, II 3:4 D, III 1:5 D, IV absent. Anterior tibiae without scopula. STC with numerous teeth in two lateral rows. Tibial apophysis with 7-10 prolateral apical spines (Fig. 4D). Palpal tibia with 2 dorsal long thin setae. Palpal organ spermophor very sinuous (Figs 4A–C). Four spinnerets, PMS short monoarticulated, PLS triarticulated apical article short, domed. Spigots without pumpkin-like socket. Cephalothorax, legs dorsally light brown, and ventrally dark brown; abdomen lighter with dark brown pattern.

**Table 6.** Length of legs palpal segments of the holotype male of *Chaco costai*.

	**Fe**	**Pa**	**Ti**	**Mt**	**Ta**	**Total**
Palp	3.9	1.9	2.4	–	1.2	9.4
I	6.7	3.8	4.7	5.5	3.8	24.5
II	5.9	3.5	4.4	5.3	3.8	22.9
III	5.0	3.2	3.8	5.7	4.4	22.1
IV	7.2	3.5	6.1	8.0	4.9	29.7

**Table 7.** Spination of legs and palps of male *Chaco costai*. The formula gives the number of spines in the following order: dorsal–prolateral–retrolateral–ventral.

	**Fe**	**Pa**	**Ti**	**Mt**	**Ta**
Palp	2-0-0-0	0-2-0-0	0-4-1-0	–	0
I	8-0-0-0	0-1-0-0	0-2-2-5	0-2-1-4	0
II	9-1-0-0	0-2-0-0	0-4-0-6	2-3-1-6	0
III	8-1-0-0	0-3-1-0	4-2-0-7	9-4-3-7	1-0-0-0
IV	9-0-0-0	0-1-1-0	4-1-4-6	7-3-3-8	1-0-0-0

Female (paratype). Total length 19.9; carapace 7.1 long, 5.7 wide; caput raised; eight eyes grouped on a tubercle, AME 0.2; PME 0.17; ALE 0.36; PLE 0.35; OQ 0.63 long, 1.16 wide; clypeus 0.27; fovea slightly procurved 1.10; sternum oval, 3.4 long, 2.9 wide. Posterior sigillae sub-circular, sub-marginal. Labium sub-rectangular, 0.47 long, 0.97 wide; 3 labial cuspules; 43/36 maxillary cuspules (Fig. 2D). Chelicerae with 8 promarginal teeth; 9 retromarginal denticles; rastellum with 18 short, thick conical setae on promargin. Leg, palpal measurements in Table 8; chaetotaxy in Table 9. Tarsus I-II scopula entire, III- IV divided by a wide band of longer conical setae, metatarsus I complete, II 2:3, III–IV absent. Anterior tibiae without scopula. STC with numerous teeth in two lateral rows. ITC I-IV absent. Claw tufts absent. Palpal claw with 4 teeth in median line. Two spermathecal receptacles, single sinuous long neck; sub-espheric fundus (Fig. 4E). Four spinnerets, PMS short monoarticulated, PLS triarticulated apical article short, domed. Spigots without pumpkin-like socket. Cephalothorax, legs brown, abdomen very light brown with darker dots.

**Table 8.** Length of legs and palpal segments of the paratype female of *Chaco costai*.

	**Fe**	**Pa**	**Ti**	**Mt**	**Ta**	**Total**
Palp	3.8	1.9	2.1	–	1.8	9.6
I	4.8	3.3	2.8	2.7	2.1	15.6
II	3.3	2.2	1.7	2.2	1.8	11.2
III	3.7	2.6	1.4	2.9	2.5	13.1
IV	3.8	2.3	2.8	3.1	2.0	14

**Table 9.** Spination of legs and palps of female *Chaco costai*.

	**Fe**	**Pa**	**Ti**		**Ta**
Palp	0-1-0-0	0-4-0-0	0-1-0-9	–	0
I	2-0-0-0	0-1-0-0	0-2-0-4	0-0-0-4	0
II	1-0-0-0	0-1-0-0	2-0-0-3	1-1-0-5	0
III	2-0-0-0	0-3-1-0	1-2-2-5	1-4-3-7	0
IV	0-0-0-0	0-0-0-0	0-0-2-2	0-3-3-8	0

##### Distribution.

Only known from the type locality.

## Supplementary Material

XML Treatment for Chaco castanea

XML Treatment for Chaco costai
